# Preventing Surgery-Induced NK Cell Dysfunction Using Anti-TGF-β Immunotherapeutics

**DOI:** 10.3390/ijms232314608

**Published:** 2022-11-23

**Authors:** Marisa Market, Gayashan Tennakoon, Marlena Scaffidi, David P. Cook, Leonard Angka, Juliana Ng, Christiano Tanese de Souza, Michael A. Kennedy, Barbara C. Vanderhyden, Rebecca C. Auer

**Affiliations:** 1 Department of Biochemistry, Microbiology and Immunology, University of Ottawa, Ottawa, ON K1H8L6, Canada; 2 Cancer Therapeutics Program, The Ottawa Hospital Research Institute, Ottawa, ON K1H8L6, Canada; 3 Department of Cellular and Molecular Medicine, University of Ottawa, Ottawa, ON K1H8L6, Canada; 4 Department of Surgery, University of Ottawa, The Ottawa Hospital, Ottawa, ON K1H8L6, Canada; 5 CI3 Centre for Infection, Immunity, and Inflammation, University of Ottawa, Ottawa, ON K1H8L6, Canada

**Keywords:** cancer surgery, natural killer cells, interferon-gamma, transforming growth factor-beta, immune suppression, immunotherapy

## Abstract

Natural Killer (NK) cell cytotoxicity and interferon-gamma (IFNγ) production are profoundly suppressed postoperatively. This dysfunction is associated with increased morbidity and cancer recurrence. NK activity depends on the integration of activating and inhibitory signals, which may be modulated by transforming growth factor-beta (TGF-β). We hypothesized that impaired postoperative NK cell IFNγ production is due to altered signaling pathways caused by postoperative TGF-β. NK cell receptor expression, downstream phosphorylated targets, and IFNγ production were assessed using peripheral blood mononuclear cells (PBMCs) from patients undergoing cancer surgery. Healthy NK cells were incubated in the presence of healthy/baseline/postoperative day (POD) 1 plasma and in the presence/absence of a TGF-β-blocking monoclonal antibody (mAb) or the small molecule inhibitor (smi) SB525334. Single-cell RNA sequencing (scRNA-seq) was performed on PBMCs from six patients with colorectal cancer having surgery at baseline/on POD1. Intracellular IFNγ, activating receptors (CD132, CD212, NKG2D, DNAM-1), and downstream target (STAT5, STAT4, p38 MAPK, S6) phosphorylation were significantly reduced on POD1. Furthermore, this dysfunction was phenocopied in healthy NK cells through incubation with rTGF-β1 or POD1 plasma and was prevented by the addition of anti-TGF-β immunotherapeutics (anti-TGF-β mAb or TGF-βR smi). Targeted gene analysis revealed significant decreases in S6 and FKBP12, an increase in Shp-2, and a reduction in NK metabolism-associated transcripts on POD1. pSmad2/3 was increased and pS6 was reduced in response to rTGF-β1 on POD1, changes that were prevented by anti-TGF-β immunotherapeutics. Together, these results suggest that both canonical and mTOR pathways downstream of TGF-β mediate phenotypic changes that result in postoperative NK cell dysfunction.

## 1. Introduction

Surgery is a primary treatment modality for cancer and provides the highest chance of a cure for most solid malignancies. However, surgery also results in profound suppression of Natural Killer (NK) cell cytotoxicity and interferon (IFN)γ production, which has been linked to increased metastases and cancer recurrence [1,2]. NK cell-derived IFNγ is critical for a robust anti-tumor response as it has anti-proliferative, pro-apoptotic, and anti-angiogenic effects that inhibit tumor cell growth [3] and regulate immune cell differentiation, activation, and homeostasis [3,4]. Impaired IFNγ production may be a main contributor to postoperative formation of cancer metastases and understanding the changes in NK cell biology responsible for impaired IFNγ is essential for the development of effective perioperative immunotherapeutics for patients undergoing cancer surgery.

In humans, NK cells are identified by surface expression of CD56. Approximately 10% of peripheral blood NK cells are CD56^bright^ and are predominantly cytokine-producing, while the remaining 90% are CD56^dim^ NK cells and are cytotoxic [5]. Natural Killer cell IFNγ production is dependent upon the integration of activating and inhibitory signals received through germline-encoded receptors [6,7]. Activating signals such as pathogen-derived antigens, stress-induced ligands [8], and stimulatory cytokines (interleukin (IL)-2/12/15/18) are antagonized by the binding of self-ligands, such as human leukocyte antigen (HLA) class I [9,10] and immunosuppressive cytokines [11]. Interestingly, postoperative NK cells have impaired IFNγ secretion following stimulation with a proprietary stimulatory cytokine cocktail (NKVue^TM^) [1], *Staphalococcus aureus,* and monocyte-derived IL-12 [12]. Surgery induces a rapid but brief pro-inflammatory phase, followed by a more prolonged postoperative anti-inflammatory phase, evolutionarily hypothesized to restore homeostasis, characterized by the release of anti-inflammatory cytokines and the expansion of immunosuppressive populations [13,14]. Our group previously demonstrated that surgically stressed B16LacZ tumor-bearing mice showed a significant increase in plasma transforming growth factor (TGF)-β1, IL-5, and IL-6(15). TGF-β1 is well known to have pro-tumorigenic and anti-inflammatory properties, is pathologically upregulated as a result of tumor cell proliferation, and is a negative predictor of disease-free survival (DFS) and overall survival (OS) [15,16,17,18]. Due to TGF-β’s potent suppression of NK cells [19,20], Viel et al. [21] and Zaiatz-Bittencourt et al. [22] investigated TGF-β signaling in NK cells and reported an inhibition of mammalian target of rapamycin (mTOR) activity in response to cytokine stimulation in the presence of TGF-β. This inhibition phenocopied NK cell incubation with rapamycin, thus suggesting that TGF-β can alter NK cell phenotype and function via mTOR inhibition [21,22]. Since TGF-β is released in the postoperative period [23,24,25], we hypothesized that impaired NK cell IFNγ production may be due to altered signaling pathways as a result of soluble TGF-β. Thus, the objectives of this study were to (1) characterize the phenotype of postoperative NK cells in terms of their surface receptor expression, downstream signaling activity, and IFNγ production, (2) investigate soluble TGF-β as a potential mechanism behind postoperative NK cell dysfunction, and (3) evaluate the use of TGF-β blocking agents in preventing NK cell dysfunction using an in vitro model of surgical stress.

## 2. Results

### 2.1. Both IFNγ Production and Secretion Are Impaired in Postoperative NK Cells

**Figure 1:**
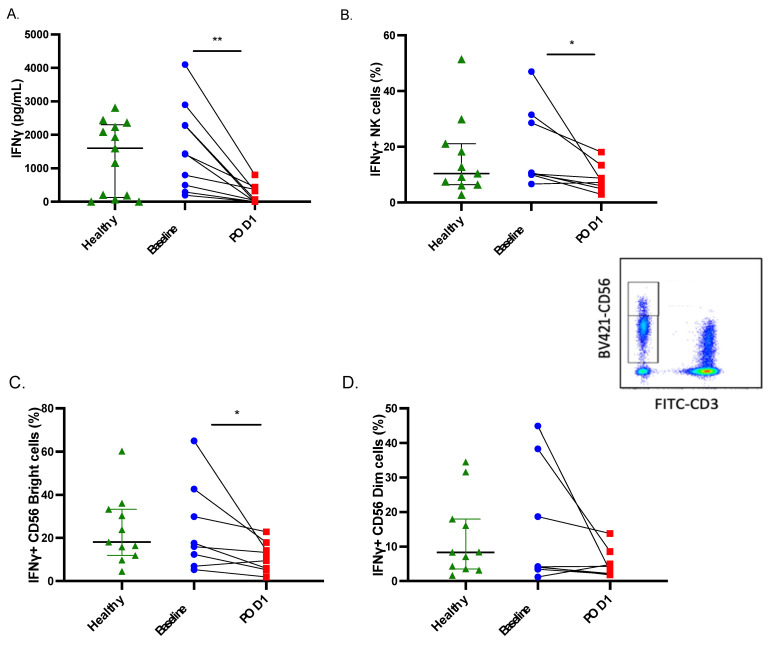
**IFNγ secretion is significantly suppressed in postoperative NK cells.** Whole blood was collected from healthy donors (green, median ± IQR shown) or patients undergoing cancer surgery at baseline (blue) and POD1 (red). Matched baseline and POD1 samples are indicated. (**A**) Whole blood was incubated for 24 h with NKVue^TM^ to stimulate IFNγ secretion, which was quantified by ELISA. Intracellular IFNγ expression (IFNγ+) was determined in (**B**) total, (**C**) CD56^bright^, and (**D**) CD56^dim^ NK cell populations following a 24 h stimulation of whole blood with recombinant interleukin (rIL)-2 (400 U/mL; Tecin^TM^ Teceleukin) and rIL-12 (20 ng/mL; R&D Systems cat#219-IL-005). * *p* ≤ 0.05, ** *p* ≤ 0.005.

Our lab has previously reported that NK cell IFNγ secretion is dramatically impaired in response to the proprietary NK cell activator Promoca^TM^ following surgery in patients with CRC [1]. Here, we sought to delineate the underlying factors contributing to this defect in a cohort of patients with various cancer types. To accomplish this, we first assessed whether the defect in IFNγ secretion could be attributed solely to impaired secretion or whether de novo production of IFNγ was impaired following cytokine stimulation. Consistent with our previous observations, we noted a significant impairment in IFNγ secretion in whole blood samples stimulated with rIL-2/12 on POD1 (*n* = 10, *p* < 0.05) (Figure 1A). Importantly, intracellular NK cell IFNγ levels were also significantly reduced on POD1 in response to stimulation with rIL-2/12 as determined by flow cytometry (*n* = 9, *p* < 0.05) (Figure 1B). Furthermore, we also analyzed CD56^Bright/Dim^CD3^−^ cell populations and found a significant reduction in the intracellular IFNγ content of CD56^Bright^ cells and a trend towards reduced intracellular IFNγ content of CD56^Dim^ cells on POD1 (*n* = 9, Figure 1C,D). Together, these results suggest that both the secretion and the de novo production of IFNγ by NK cells in response to activating cytokines is impaired in the postoperative setting.

### 2.2. Postoperative NK Cells Have Reduced Receptor Expression and Downstream Signaling Activity

**Figure 2:**
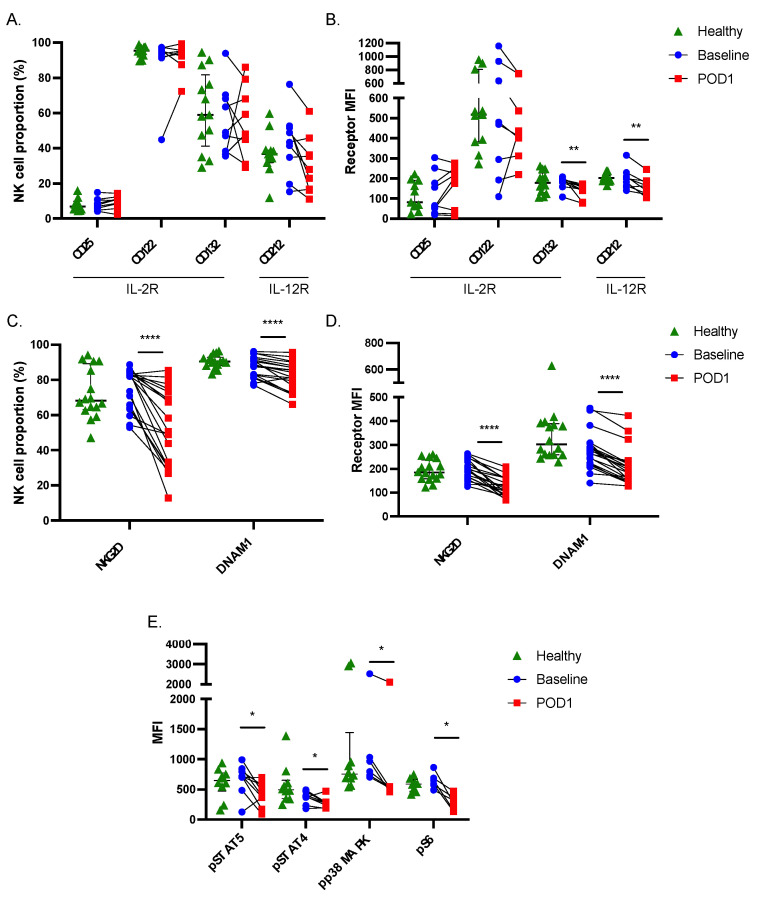
**Postoperative NK cells have significantly reduced cytokine and activating receptor expression and reduced phosphorylation of downstream signaling proteins.** Whole blood was collected from healthy donors (green, median ± IQR shown) or patients undergoing cancer surgery at baseline (blue) and POD1 (red). Matched baseline and POD1 samples are indicated. Expression (% and MFI) of IL-2R subunit CD132 and IL-12R subunit CD212 ((**A**) (%)/(**B**) (MFI)) and activating receptors NKG2D and DNAM-1 ((**C**) (%)/(**D**) (MFI)) is shown. The phosphorylation of all assessed downstream signaling proteins (pSTAT5, pSTAT4, pP38 MAPK, pS6) in response to stimulation with rIL-2/12 was significantly reduced in NK cells on POD1 (**E**). * *p* ≤ 0.05, ** *p* ≤ 0.005, **** *p* ≤ 0.00005.

Next, since NK cell responses to rIL-2/12 were impaired, we first investigated the effects of surgical stress on the surface expression of activating cytokine receptor subunits. These included CD25, CD122, and CD132, which comprise the high affinity IL-2R and CD212, a constitutively expressed receptor subunit for IL-12. In agreement with the impaired responsiveness of NK cells to rIL-2/12 stimulation postoperatively, we observed a significant reduction in CD132 median fluorescence intensity (MFI) and CD212 MFI expression on POD1 (*n* = 9, *p* < 0.05) (Figure 2A,B).

NK cell activity is tightly regulated by the integration of both activating and inhibitory signals through additional germline-encoded receptors. When the activating signals outweigh the inhibitory signals, this tips the balance towards NK cell activation. Thus, we sought to determine whether these observed changes extended to the expression of two additional activating receptors (NKG2D and DNAM-1) and three inhibitory receptors (NKG2A, PD-1, and TIGIT)(29). We found that surgically stressed NK cells expressed significantly less NKG2D, DNAM-1, NKG2A, and TIGIT (*n* = 20, *p* < 0.05), with the most profound and consistent reductions being observed in NKG2D and DNAM-1 expression (Figure 2C,D and Supplemental Figure S1). Taken together, these data suggest that NK cell dysfunction during the postoperative period is correlated with a reduction in expression of several activating receptors, relative to the inhibitory receptors, tipping the balance towards an inactivated state.

### 2.3. Signal Transduction in Response to Cytokine Stimulation Is Impaired in Postoperative NK Cells

Ligation of IL-2 and IL-12 with their respective receptors is known to induce Janus kinase (JAK)/Signal Transducer and Activator of Transcription (STAT) signaling, which results in the phosphorylation of STAT5 and STAT4, respectively, leading to IFNγ production. Both cytokines are also known to regulate IFNγ through JAK/STAT-independent signaling pathways as well. Engagement of the IL-2R results in signaling through the Phosphoinositide 3-kinase (PI3K)/Akt pathway, which potentiates mammalian target of rapamycin complex 1/2 (mTORC1/mTORC2) formation, affecting transcriptional machinery to control cell growth and proliferation, specifically the activity of the ribosomal protein S6 through the activity of S6K [26,27,28,29,30]. IL-12 activates p38 MAPK to stabilize IFNγ mRNA [31]. To determine whether the previously noted reductions in receptor expression resulted in reduced signaling in response to NK cell stimulation, we assessed the phosphorylation status of STAT5 (*n* = 9), STAT4 (*n* = 9), p38 MAPK (*n* = 7), and S6 (*n* = 6) by flow cytometry. Without exception, all patients displayed significant reductions in the phosphorylation of these signaling proteins on POD1 (*p* < 0.05) (Figure 2E). Therefore, impaired IFNγ production in surgically stressed NK cells may be a consequence of the downregulation of upstream receptors and impaired intracellular transduction of activating signals.

### 2.4. NK Cell Suppressive Factors Are Present in Postoperative Patient Plasma

**Figure 3:**
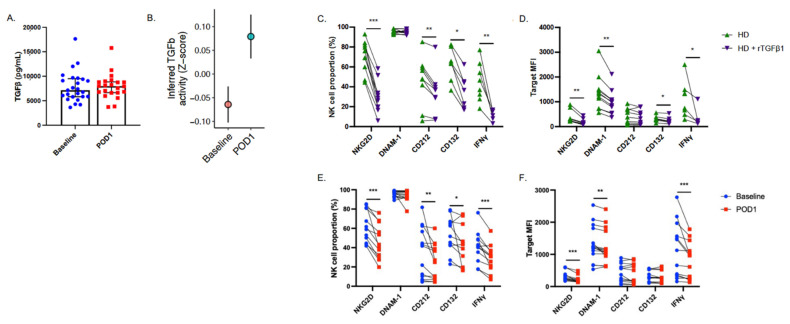
**Postoperative NK cells show increased TGF-β and POD1 plasma induces NK cell dysfunction in vitro.** (**A**) Platelet-free plasma was isolated by centrifugation from whole blood from 25 baseline and POD1 patients undergoing cancer surgery. TGF-β was quantified by ELSA. Median ± IQR is shown (*p* = ns). (**B**) Single-cell RNA sequencing in six patients with colorectal cancer underwent TGF-β activity analysis. Median ± IQR is shown. Flow cytometry staining showing expression, (**C**) %, and (**D**) MFI of NK activating receptors, NKG2D, DNAM-1, CD212, CD132, and intracellular IFNγ in NK cells isolated from health donors incubated in the absence (green) or presence (purple) of recombinant TGF-β (10 ng/mL). Healthy donor isolated NK cells were also incubated with baseline (blue) or POD1 (red) plasma and expression, (**E**) %, and (**F**) MFI of the same targets as (**C**,**D**) were determined. * *p* ≤ 0.05, ** *p* ≤ 0.005, *** *p* ≤ 0.0005.

The postoperative period is associated with the systemic release of numerous anti-inflammatory factors which can be detected in patient plasma [32]. As stated above, TGF-β has been shown to increase postoperatively and is associated with impaired NK cell effector functions. We observed an overall increase in plasma TGF-β in 25 patients undergoing cancer surgery from median 7.15 ng/mL to 7.97 ng/mL on POD1 (Figure 3A and Supplemental Table S3). We also observed a significant increase in TGF-β signaling activity (*p* = 0.016) in POD1 NK cells via scRNA-seq (Figure 3B). We next sought to determine whether NK cell dysfunction could be attributed to soluble TGF-β present in postoperative patient plasma. To accomplish this, healthy isolated CD56^+^ cells were incubated in complete media containing low-dose rIL-2 and supplemented with either healthy plasma +/− rTGF-β1 (used as a positive control) or baseline or POD1 plasma from CRC surgery patients for 24 h (Supplemental Table S2). Cells were then stained for receptor expression (NKG2D (rTGF-β1 *n* = 10, POD1 plasma *n* = 12), DNAM-1 (rTGF-β1 *n* = 11, POD1 plasma *n* = 15), CD212 (rTGF-β1 *n* = 9, POD1 plasma *n* = 13), and CD132 (rTGF-β1 *n* = 7, POD1 plasma *n* = 1)). In parallel, cultures were stimulated with activating concentrations of rIL-2/12 to assess intracellular IFNγ production (rTGF-β1 *n* = 8, POD1 plasma *n* = 13).

Incubation with healthy plasma spiked with rTGF-β1 resulted in significant reductions in the expression of CD212 %, CD132 %, NKG2D % and MFI, DNAM-1 MFI, and IFNγ % and MFI production following rIL-2/12 stimulation, as compared to cells cultured with healthy plasma alone (*p* < 0.05) (Figure 3C,D). In this culture system, rTGF-β reproduced the dysfunctional postoperative NK cell phenotype. Furthermore, the addition of POD1 plasma was sufficient to significantly reduce expression of CD212 %, CD132 %, NKG2D % and MFI, DNAM-1 MFI, and IFNγ % and MFI production (*p* < 0.05) (Figure 3E,F). Together, these findings suggest that the presence of one or more soluble factors in POD1 patient plasma is able to induce NK cell dysfunction alone. These results, in combination with our scRNA-seq findings which point towards upregulated TGF-β signaling in POD1 NK cells, have led us to hypothesize that this soluble factor is TGF-β.

### 2.5. Targeted Inhibition of TGF-β Prevents NK Cell Dysfunction When Cultured with Postoperative Plasma

**Figure 4:**
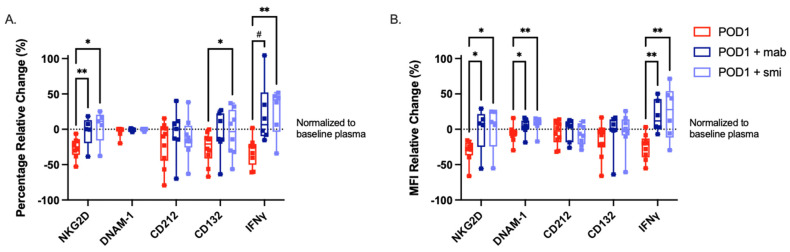
**Anti-TGF-β therapies prevent POD1 plasma-induced NK cell dysfunction.** Patient POD1 plasma (red) was pre-incubated with an anti-TGF-β monoclonal antibody (mAb) or isolated healthy NK cells were pre-incubated with the TGF-βR1 small molecule inhibitor (smi) SB525334. Box plot of NK cell activating receptor expression and IFNγ production ((**A**) (%)/(**B**) (MFI)) normalized to incubation in baseline plasma. Median ± IQR is shown. ^#^ *p* ≤ 0.1, * *p* ≤ 0.05, ** *p* ≤ 0.005.

Next, we sought to determine whether targeting TGF-β could prevent the NK cell dysfunction observed following incubation with POD1 plasma. We prevented the engagement of TGF-β with its receptor using a monoclonal antibody and the TGF-βR subunit 1 (ALK5) or a small molecule inhibitor (SB525334). We assessed the expression of NKG2D (mAb *n* = 5, smi *n* = 5), DNAM-1 (mAb *n* = 8, smi *n* = 8), CD212 (mAb *n* = 7, smi *n* = 8), CD132 (mAb *n* = 7, smi *n* = 8), and intracellular IFNγ (mAb *n* = 6, smi *n* = 6). The addition of the mAb was sufficient to maintain the expression of NKG2D %, DNAM-1 MFI, and production of IFNγ % and MFI following incubation with POD1 patient plasma. Likewise, the addition of the smi was sufficient to maintain the expression of NKG2D % and MFI, DNAM-1 MFI, CD132 %, and production of IFNγ % and MFI following incubation with POD1 patient plasma (Figure 4A,B). Together, these results implicate TGF-β as a mediator of postoperative NK cell dysfunction.

### 2.6. Single-Cell RNA Sequencing Reveals Impaired Metabolism in Surgically Stressed NK Cells

**Figure 5:**
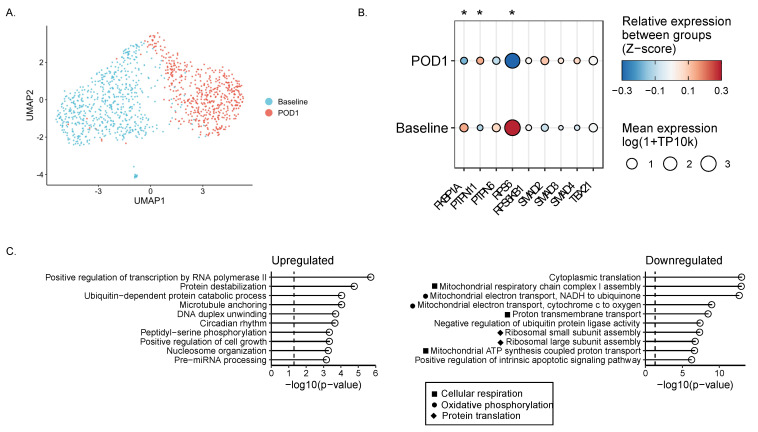
**NK cells exhibit dysregulated metabolism and reduced mRNA translation on POD1.** Six matched baseline and POD1 patient samples were collected and processed for multiplexed scRNA-seq with the 10× Genomics Chromium technology. (**A**) UMAP plot of NK cell scRNA-seq data at baseline (blue) and POD1 (red). Each point corresponds to a single cell. (**B**) Heat map of average expression levels of canonical and non-canonical genes downstream of TGF-β genes in NK cells at baseline and POD1. TP10k = transcripts per 10 k transcripts. (**C**) Gene set enrichment analysis (GSEA), showing a list of the top significant upregulated and downregulated pathways. * *p* ≤ 0.05.

Single-cell RNA sequencing was performed on cryopreserved PBMCs from six patients with colorectal cancer having surgery recruited to the PERIOP-02 clinical trial. Based on our previous findings highlighting the dysfunction of postoperative NK cells, we focused our analysis on only the NK cell population and were able to visualize changes in the gene expression profiles associated with POD1 samples (Figure 5A). We found 163 significantly downregulated genes and 218 significantly upregulated genes on POD1 as compared to baseline (*p* < 0.03). Targeted gene analysis revealed significant postoperative decreases in S6 (*RPS6*) and FKBP12 (FKBP1A) and a significant postoperative increase in Shp-2 (*PTPN11*) gene expression (*p* < 0.05) (Figure 5B).

TGF-β is thought to inhibit NK cell function via canonical and non-canonical pathways. In the canonical pathway, transcription factor Smad2/3 is activated, translocates to the nucleus, and complexes with Smad4 to inhibit IFNγ production via direct binding of the T-bet (*Tbx21*) promoter [21,33,34]. The non-canonical pathways by which TGF-β1 signals in NK cells remain largely uncharacterized, although TGF-β has been shown to inhibit mTOR activity in NK cells [21]. Inhibition of mTOR by TGF-β1 is rapid and concomitant with Smad2/3 phosphorylation [21]. Independent of Smad signaling, FKBP12 constitutively binds to TGF-βRI [35], inhibiting signal propagation in the absence of TGF-β. Interestingly, FKBP12 can inhibit mTOR activity in complex with its endogenous binding partners or rapamycin [36]. In T cells, TGF-β1 upregulates the expression of and activates protein tyrosine phosphatase (PTP) Src homology region 2 domain-containing phosphatase-1/2 (Shp-1, Shp-2), which inhibits the activity of signaling kinases [37,38]. Shp-1 activity has been shown to mediate mTOR function in NK cells via intracellular propagation of inhibitor receptor signals [39,40], although to date there are no reports of Shp-1 activity downstream of TGF-β1 signaling in NK cells. mTOR kinase activity functions as a “molecular rheostat” to control NK cell metabolism and activity [40]. We identified significant postoperative decreases in genes associated with canonical mTORC1 functions including cellular respiration, oxidative phosphorylation, and protein translation (Figure 5C). Basal metabolic activity (specifically oxidative phosphorylation) is necessary for NK cell metabolic fitness, degranulation, and IFNγ secretion in response to activating receptor ligation and stimulatory cytokines in both mice and humans [41]. As a consequence, impaired mTOR signaling may play a role in the profound dysfunction of postoperative NK cells [42].

### 2.7. Smad2/3 Phosphorylation Is Increased While S6 Phosphorylation Is Reduced in Postoperative NK Cells

**Figure 6:**
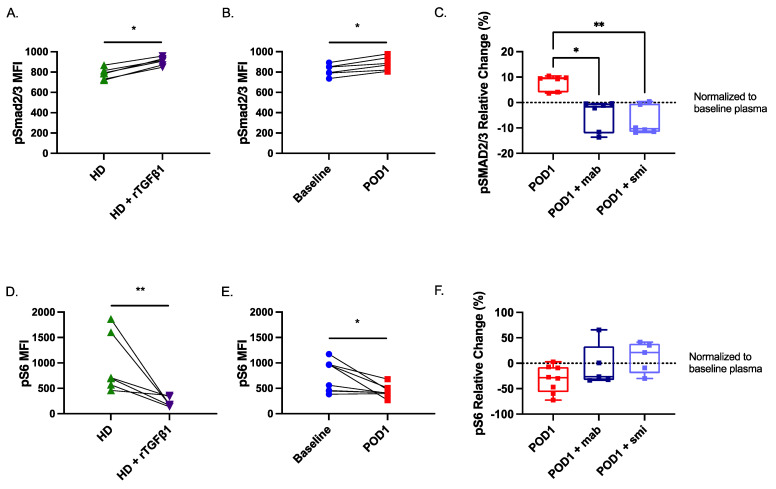
**TGF-β signaling via SMAD2/3 phosphorylation is increased on POD1 in NK cells, which coincides with decreased S6 phosphorylation.** Flow cytometry staining of SMAD2/3 phosphorylation in isolated healthy NK cells following addition of (**A**) rTGF-β (10 ng/mL) and (**B**) POD1 plasma. (**C**) Box plot (median ± IQR) of relative change in SMAD2/3 phosphorylation following TGF-β blockade with anti-TGF-β monoclonal antibody (mAb, 100 μg/mL) or TGF-βR1 small molecule inhibitor (smi, 2 μM) SB525334 in isolated healthy NK cells, normalized to incubation with baseline plasma. S6 phosphorylation in isolated healthy NK cells following incubation in (**D**) rTGF-β and (**E**) POD1 plasma. (**F**) Box plot (median ± IQR) of relative change in S6 phosphorylation following TGF-β blockade with anti-TGF-β mAb or TGF-βR1 smi SB525334 in isolated healthy NK cells, normalized to incubation with baseline plasma. * *p* ≤ 0.05, ** *p* ≤ 0.005.

Our scRNA-seq dataset suggests that Smad2 signaling is increased while mTORC1 signaling is suppressed in NK cells on POD1. Thus, we assessed the potential role of both canonical (Smad2/3-mediated) and non-canonical (mTOR-mediated) signaling pathways in the observed NK cell dysfunction. To investigate this hypothesis, we first assessed changes in the phosphorylation of Smad2/3, a direct downstream signaling target from the TGF-β receptor. We then assessed the phosphorylation of the S6 protein, a well-established surrogate readout for mTORC1 kinase activity [21]. Consistent with our hypothesis, we observed a significant increase in pSmad2/3 (*n* = 6) in healthy NK cells cultured in the presence of rTGF-β1 or POD1 plasma (Figure 6A,B). The addition of the TGF-β mAb (*n* = 6) and smi (*n* = 6) significantly inhibited the phosphorylation of Smad2/3 (*p* < 0.05) (Figure 6C). We also observed a significant reduction in pS6 (*n* = 8) in healthy NK cells cultured in the presence of rTGF-β1 or POD1 plasma (Figure 6D,E). Finally, the addition of the TGF-β mAb (*n* = 5) and smi (*n* = 5) prevented the reduction in S6 phosphorylation (Figure 6F). In summary, these results suggest that, in the postoperative period, soluble TGF-β can induce NK cell dysfunction via canonical and non-canonical pathways. Moreover, blocking TGF-β signaling alone is sufficient to restore mTORC1 activity.

## 3. Materials and Methods

### 3.1. Clinical Protocols

#### 3.1.1. Perioperative Human Blood and Tissue Specimen Collection Program (PHBSP) Protocol No. 2011884-01H

This protocol was approved by the Ottawa Health Science Network Research Ethics Board. All subjects gave written informed consent in accordance with the Declaration of Helsinki. Eligible patients were >18 years of age and had a planned surgical resection of a primary or metastatic tumor (cancer patients) or were healthy donors who volunteered to participate. Exclusion criteria included a history of active viral or bacterial infection, known HIV or Hepatitis B or C, autoimmune diseases, or use of immunosuppressive medications. The primary objective was to immunophenotype NK cells from healthy donors and cancer patients prior to surgery (baseline) and after surgery on postoperative day (POD) 1.

#### 3.1.2. Single-Cell RNA Sequencing Patient Sample Protocol (NCT02987296)

Baseline, POD1, and healthy donor peripheral blood samples (20–40 mL) were drawn at The Ottawa Hospital after receiving approved informed consent under the following clinical protocols: (i) OHSN-REB# 20160732-01H and (ii) OHSN-REB# 2011884-01H. Patients were instructed to take supplemental arginine (*n* = 3) or an isocaloric/isonitrogenous control supplement (*n* = 3) three times per day for 5 days as part of the trial. The primary objective of the clinical study was to characterize human surgery-induced (sx) myeloid-derived suppressor cells (MDSCs) for the purpose of identifying a pathway amenable to therapeutic targeting.

### 3.2. Patient Demographics

NK cell phenotype and function were assessed using whole blood collected from 42 healthy donors and 39 patients undergoing cancer surgery (Supplemental Table S1). Patient populations were representative of different cancer types and disease burden, highlighting the broad applicability of our findings. Plasma transfer assays utilized NK cells (CD56^+^) isolated from 18 healthy donors, and plasma from 27 healthy donors and 58 cancer patients at baseline and on POD1 (Supplemental Table S2). Single-cell RNA sequencing utilized cryopreserved samples of peripheral blood mononuclear cells (PBMCs) from six patients with colorectal cancer (CRC).

### 3.3. Antibodies and FACS Analysis

The monoclonal antibodies used are summarized in Table 1. Fluorescence cytometry data was acquired with a BD LSRFortessa using BD FACSDiva^TM^ version 8.0.3 software (BD Biosciences, San Diego, CA, USA) and data were analyzed with FlowJo, LLC version 10.6.1 software (FlowJo LLC, Ashland, OR, USA).

### 3.4. Whole Blood Phenotyping and Functionality Assays

Whole blood was collected from healthy donors or patients undergoing cancer surgery at baseline and POD1 and stained for intracellular and extracellular targets using the previously published protocols [43]. Briefly, whole blood was either stained immediately for extracellular activating/inhibitory receptors (NKG2D, DNAM-1, NKG2A, PD-1, TIGIT) or cytokine receptor subunits (CD25, CD122, CD132, CD212) or incubated without stimulation (control) or with recombinant interleukin (rIL)-2 (400 U/mL; Tecin^TM^ Teceleukin) and rIL-12 (20 ng/mL; R&D Systems cat#219-IL-005). Plasma was collected to quantify extracellular cytokines by ELISA or red blood cells were lysed and cells were stained for intracellular IFNγ or intracellular phosphor-signaling molecules (STAT5, STAT4, p38 MAPK, S6). Samples were acquired by flow cytometry and target expression was assessed on live CD56^+^CD3^−^ populations.

### 3.5. Platelet-Free Plasma Collection

Blood was collected in sodium-heparin tubes from healthy donors and patients undergoing cancer surgery at baseline and POD1. Whole blood was centrifuged at 1000× *g* for 10 min, and plasma was collected and centrifuged again at 10,000× *g* for 15 min to collect platelet-free plasma. Platelet-free plasma was stored at −80 °C to be used either for quantification of protein by ELISA or for plasma transfer assays.

### 3.6. ELISA

Human IFNγ and TGF-β (Supplemental Table S3) were quantified using the R&D Quantikine^®^ ELISA immunoassay according to the manufacturer’s specifications. Samples were thawed at room temperature (RT) prior to analysis.

### 3.7. Cell Isolation

Approximately 1e6 CD56^+^ cells per 10 mL of whole blood were isolated using the StraightFrom^TM^ Whole Blood CD56^+^ magnetic bead isolation kit (Miltenyi cat #130-090-875) via a Miltenyi AutoMACS^TM^ cell sorter.

### 3.8. Combined Plasma Culture Assays: Measuring Effects of Patient Plasma on NK Cell Function and Phenotype Ex Vivo

Platelet-free plasma from multiple healthy, baseline, or POD1 samples were combined per assay as master mixtures to ensure sufficient volume and consistency, hereafter referred to as healthy, baseline, or POD1 plasma for simplicity. CD56^+^ cells were isolated from healthy whole blood. Average viability was > 80% and average purity was >90%. Isolated CD56^+^ cells were plated at 1.5 × 10^5^ cells/well and incubated for 24 h at 37 °C in 200 µL of RPMI supplemented with 2% Fetal Bovine Serum (FBS) (GE Healthcare cat#SH3039603), low-dose rIL-2 (100 U/well), and 25% (50 μL) healthy, baseline, or POD1 plasma. When indicated, CD56^+^ cells were also incubated in the presence of rTGF-β1 (10 ng/mL) as a positive control in 200 μL 2% FBS-supplemented RPMI with 25% healthy plasma for 24 h. When indicated, plasma was incubated for 30 min at 37 °C with 100 μg/mL of anti-TGF-β mouse monoclonal antibody (clone 1D11, Bio X Cell cat#BE0057) prior to culture with CD56^+^ cells. When indicated, isolated CD56^+^ cells were incubated with the TGF-β receptor subunit 1 (ALK5) small molecule inhibitor (smi) SB525334 (Selleck Chemicals) (2 μM/well) for 1 h at 37 °C prior to adding plasma.

At 24 h of incubation, cells were stained for surface receptors or intracellularly for phosphorylated S6 (pS6; Ser235/236). At 6 h, cells were stimulated with rIL-2 (400 U/well) and rIL-12 (20 ng/well) for the remaining 18 h to assess IFNγ production.

### 3.9. In Vitro Staining Protocol

NK cells were stained for surface receptors NKG2D, DNAM-1, CD212, and CD132. After centrifuging at 500× *g* for 5 min, cells were resuspended in 40 μL of extracellular staining (ECS) mix and incubated at 4 °C for 20 min in the dark. An amount of 200 μL of flow buffer (FB; PBS + 2.5g BSA + 0.5M EDTA) was added prior to centrifuging at 500× *g* for 5 min. Cells stained for extracellular receptor targets were then resuspended in 200 μL 1% paraformaldehyde (PFA) and stored at 4 °C for up to 72 h prior to acquisition and analysis by flow cytometry.

NK cells stained for intracellular pS6 were resuspended in 200 μL pre-warmed BD Cytofix (BD biosciences cat#554655) and incubated for 10 min at 37 °C. The plate was centrifuged at 500× *g* for 5 min and wells were resuspended in 100 μL chilled BD Perm III buffer (BD biosciences cat#558050) and incubated for 30 min at 4 °C. An amount of 150 μL of FB was added prior to centrifuging at 500× *g* for 5 min. Cells were washed once with 200 μL of FB, resuspended in 100 μL intracellular staining (ICS) mix, and incubated at 4 °C for 1 h. The plate was centrifuged at 500× *g* for 5 min and cells were resuspended in 200 μL 1% PFA and stored at 4 °C prior to acquisition and analysis by flow cytometry.

NK cells used to assess intracellular IFNγ production were centrifuged at 500× *g* for 5 min and resuspended in control media (RPMI + 2% FBS + 100U/well rIL-2) or rIL-2/12 stimulation media (2% RPMI + 2% FBS + 400U/well rIL-2 + 20ng/well rIL-12) and incubated at 37 °C for 24 h. BD Golgiplug^TM^ (Brefeldin A, BD biosciences cat#555029) was added (2 μL/well) for the remaining two hours of incubation. The plate was centrifuged at 500× *g* for 5 min, decanted, and washed with 200 μL of FB prior to resuspension in 40 μL of ECS mix. Cells were incubated at 4 °C for 20 min, 100 μL of FB was added to each well, and the plate was centrifuged again at 500× *g* for 5 min. Cells were resuspended in 200 μL IC fixation buffer (Invitrogen cat#00-8222-49) and incubated at RT for 30 min. Cells were centrifuged at 500× *g* for 5 min, resuspended in chilled BD Perm III Buffer, and incubated at 4 °C for 30 min in the dark. Cells were washed once with 100 μL of FB, resuspended in 100 μL of ICS mix, and incubated at 4 °C for 30 min in the dark. The plate was centrifuged at 500× *g* for 5 min and cells were resuspended in 200 μL 1% PFA and stored at 4 °C for up to 72 h prior to acquisition and analysis by flow cytometry.

### 3.10. Single-Cell RNA Sequencing

PBMCs were isolated by Ficoll-Paque density centrifugation from blood collected from patients with CRC having surgery recruited to the PERIOP-02 (NCT02987296) clinical trial. All six patients were male, 3/6 patients were 60–69 years old, and 4/6 patients had Stage II colorectal cancer. The cells were cryopreserved in liquid nitrogen in freezing media (90% FBS and 10% DMSO). Baseline and POD1 samples from six patients were labelled with a unique lipid-modified DNA barcode [44] to enable multiplexed scRNA-seq while a parallel sample was processed for immunophenotyping and viability assessment by flow cytometry. The individually labeled baseline and POD1 samples were separately pooled and processed for scRNA-seq using the 10× Genomics 3′ RNA-seq platform (v3; StemCore, Ottawa, ON, Canada). Barcode and cDNA libraries were prepared separately, as outlined by McGinnis et al. [44], and sequenced on an Illumina NextSeq500 to a depth of >20,000 reads per cell. Gene expression libraries were aligned to the GRCh38 build of the human genome using cellranger v3.1.0. Individual cells from the pooled data were assigned to specific samples by aligning sequencing reads to the list of possible barcode sequences using the deMULTIplex R package. The dataset for this study can be made available upon reasonable request.

### 3.11. Statistics

Descriptive statistics were used to summarize data collected on extracellular receptors, phospho-signaling proteins, and IFNγ production (median with interquartile range (IQR)). The Wilcoxon matched-pairs signed rank test was used to determine if there were significant changes in target protein expression/secretion between baseline and POD1 samples. One-way ANOVA with Geisser–Greenhouse correction or two-way ANOVA with Dunnett correction for multiple comparisons were used to determine if there were significant changes in target protein expression between conditions in plasma transfer experiments. All statistical analyses were determined using GraphPad Prism version 8.0 software (La Jolla, CA, USA). The level for statistical significance was set a priori at ≤ 0.05 (* *p* ≤ 0.05, ** *p* ≤ 0.005, *** *p* ≤ 0.0005, **** *p* ≤ 0.00005).

## 4. Discussion

Surgery is necessary for curative intent in most patients with solid tumors. However, the majority of cancer-related deaths are attributed to postoperative metastasis [45,46]. Thus, despite the many advancements in cancer treatment, remission rates have plateaued, due mostly to our inability to effectively eradicate micrometastases [2,47]. Natural Killer cells are critical for the anti-tumor response and postoperative NK cell suppression (dysfunctional cytotoxicity and cytokine secretion) has been correlated with increased metastases in animal models [2,48,49]. In human studies, reduced NK cell activity is associated with increased rates of cancer recurrence and death [50,51,52]. Therefore, there is an urgent need to understand postoperative NK cell suppression and its contribution to cancer recurrence to prevent the formation of postoperative metastases.

Our data suggest that suppressed IFNγ production by NK cells following invasive surgery can be attributed to reduced activating and cytokine receptor expression (CD212, CD132, NKG2D, and DNAM-1) and impaired phosphorylation of signaling proteins (STAT4, STAT5, p38 MAPK, and S6). This is the first study to show reduced activating receptor expression and impaired downstream phosphorylation in postoperative NK cells. Our lab has previously shown that postoperative NK cells from CRC surgery patients have reduced IFNγ secretion in response to cytokine stimulation using NKVue^TM^ [1], a proprietary cytokine stimulatory cocktail. The most profound suppression was observed on POD1, where we saw 90.2% (37/41) of patients had IFNγ levels below the minimum detectable level [1]. A similar phenotype of suppressed IFNγ has been observed in the CD56^bright^ NK cell population by Reinhardt and colleagues in response to IL-12 stimulation. They found a significant decrease in CD212 expression and impaired IFNγ production up to POD7 in non-cancer surgery patients [12]. The results of this study suggest that this suppressed postoperative phenotype is due to the downstream effects of TGF-β on NK cells following surgery.

TGF-β has been shown to dampen NK cell proliferation, cytotoxicity, and IFNγ secretion [19,21,53]. In culture, TGF-β can override IL-2 activation of NK cells and induce the downregulation of activating receptors, including NKG2D and DNAM-1 [19,20,54,55,56]. In addition to the downregulation of NK cell receptors, Viel et al., showed that treatment with TGF-β and rapamycin both opposed the IL-15-induced phosphorylation of mTORC1 substrates S6 and 4EBP1 and mTORC2 substrate Akt in vitro, resulting in mTORC-dependent inhibition of NK cell cytotoxicity and IFNγ secretion [21]. In vivo, NK cell development was arrested by TGF-β signaling or mTOR depletion, while mTOR activity and NK cell cytotoxicity were enhanced by TGF-βRII depletion [21]. Taken together, this indicates a critical role for mTOR in mediating the effects of TGF-β1 in NK cells. Viel et al., also showed that TGF-β1-mediated inhibition of mTOR was rapid (1 h) and concomitant with Smad2/3 phosphorylation [21]. Furthermore, TGF-β is well known to inhibit IFNγ production through canonical Smad2/3-dependent repression of “master regulator” transcription factor T-bet [21,33,34]. Our results suggest that postoperative soluble TGF-β acts to induce NK cell suppression via both canonical and non-canonical (mTOR inhibition) pathways, which may act to impair NK cell metabolism.

ScRNA-seq revealed a reduction in RNA expression associated with critical metabolic processes, including oxidative phosphorylation (OxPhos) and cellular respiration. Acutely, NK cell functions rely predominantly on OxPhos; however, chronic stimulation with cytokines produces a shift towards glycolysis to meet energy requirements [57]. mTOR is well known to be an important regulator of cell growth and metabolism [26,31]. Specifically, mTORC1 activity is necessary for activated NK cell glycolysis and IFNγ production, which can be inhibited by rapamycin [58]. Viel et al., assessed cell growth, glycolysis, OxPhos, and nutrient transporter expression (CD71 and CD98) in response to IL-15 in murine splenic NK cells and primary human NK cells and found that TGF-β and rapamycin were equally efficient at inhibiting NK cell metabolic activities, suggesting a critical role for non-canonical mTOR inhibition in response to TGF-β [21]. However, Bittencourt and colleagues recently published that TGF-β only inhibited mTORC1 after sustained (5 days) stimulation of human NK cells with IL-2 and not at earlier time points. Furthermore, they suggest that the inhibition of early metabolic changes (OxPhos, glycolysis, and CD71 expression) occurs downstream of the canonical pathway only [22]. Our results suggest that postoperative TGF-β acts via both canonical and non-canonical pathways to suppress NK cell receptor expression and IFNγ production. Using NK cells from patients with breast cancer, Slattery and colleagues showed that these cells had reduced IFNγ production, cytotoxicity, and reduced rates of OxPhos and glycolysis compared to healthy NK cells. These functional and metabolic impairments were reversed when the NK cells were cultured ex vivo with an anti-TGF-β mAb [59]. Thus, future studies will aim to further explore the metabolic profile of postoperative NK cells.

In addition to showing that postoperative TGF-β impairs NK cell function on POD1, here we show that this suppression can be prevented in vitro with TGF-β-specific immunotherapeutics (anti-TGF-β mAb and ALK5 smi SB525334), thus suggesting postoperative TGF-β as a potential perioperative therapeutic target. Despite these promising results in preclinical cancer models, there is a paucity of literature assessing TGF-β blockade in the postoperative period. Furthermore, targeting TGF-β may have adverse effects in the context of cancer and in surgery related to wound healing [60,61]. TGF-β’s role in wound healing is complex as it can both stimulate and protract wound re-epithelialization [62,63]. Additionally, TGF-β is critical for maintaining immune homeostasis and preventing autoimmunity via the promotion of suppressive cell populations and inhibition of immune cell proliferation/activity. This is evident in TGF-β- or TGF-βR-deficient mice that rapidly succumb to systemic T cell-mediated autoimmunity [11,19,21,53,54,55,56]. Future studies will aim to investigate the systemic consequences of blocking TGF-β perioperatively using our murine model of surgical stress, the protocols for which have been described previously [2,64].

There are two possible avenues to target postoperative TGF-β: targeting production or targeting TGF-β signaling in NK cells. Possible postoperative sources of TGF-β include activated platelets and (sx)MDSCs. Almost half of basal plasma TGF-β is believed to derive from platelets [65]. Activated platelets not only release latent TGF-β in α granules, but also activate this latent form via a furin-like proprotein convertase [66,67]. In the postoperative period, activated platelets contribute to a hypercoagulable state [64,68], which has been reported to potentiate metastases and prevent NK cell recognition of tumor cells via peritumoral clot formation [64]. Outside of the postoperative period, platelet-induced NK cell dysfunction has been well documented. Studies investigating NK cell activity in models of endometriosis and cancer metastasis support multiple mechanisms by which platelets may inhibit NK cell function. These include the physical coating of target cells (termed “peritumoural clots” [64] or “pseudo-self-cloaks” [69,70]) as a shield against NK cell-mediated cytotoxicity [71], as well as TGF-β-mediated target cell upregulation of self-ligands/downregulation of stress-induced ligands, and aberrant regulation of NK cell activating and inhibitory receptors [19,69,70,72,73,74]. Postoperatively, a hypercoagulable state and activated platelets have been shown to impair NK cell-mediated anti-tumor functions [64]. Thus, therapies aimed at targeting the platelet release of TGF-β, including anti-GP1bα mAbs, may prove beneficial for postoperative NK cell function. However, since platelet activation and aggregation are critical for clot formation and wound healing [67], inhibition of platelet functions postoperatively may worsen bleeding diathesis and impair post-surgical recovery.

Evolutionarily, myeloid cells are important host protectors, acting to prevent infection and aid in tissue remodeling [75]. Notably, surgery acts as a form of trauma which can trigger emergency myelopoiesis and MDSC accumulation in both murine models [76,77,78] and humans [1,79,80]. MDSCs are a heterogeneous population of immature myeloid cells and have been shown to promote cancer growth and metastasis. In many cancers, high circulating MDSC numbers can be inversely correlated with clinical response and poor prognosis [77,78,81,82]. There are many potential mechanisms of MDSC-mediated NK cell suppression that may contribute to immune tolerance and metastasis, including via secreted or membrane-bound TGF-β [13,23,83,84].

Given the ability of MDSCs to secrete TGF-β, this may underline an autocrine positive feedback loop which further bolsters postoperative MDSC accumulation and immunosuppressive activity [85]. Interestingly, TGF-β promotes the C/EBP transcription factor, a hallmark regulator of MDSC activity, suggesting that TGF-β’s effects occur at a transcriptional level [86]. Li et al., demonstrated that membrane-bound TGF-β can significantly impair NK cell cytotoxicity, NKG2D, and IFNγ expression in vitro. Physical separation of NK cells and MDSCs in co-culture by a transwell abrogated the observed MDSC-mediated NK cell suppression, highlighting the contact-dependent nature of this interaction in this context [23]. Due to their overall suppressive function, (sx)MDSCs are an exciting perioperative target to prevent NK cell suppression. However, depleting this population could be detrimental by enabling uncontrolled inflammation, further contributing to postoperative complications. As future research delineates the significance of (sx)MDSC-derived TGF-β, it is important to keep their evolutionary homeostatic role in mind.

Given that platelets, (sx)MDSCs, and TGF-β all play important roles in postoperative wound healing, homeostasis, and prevention of autoimmunity, targeting the effects of TGF-β in NK cells directly may be the best avenue by which to prevent postoperative suppression. Specifically, the genetic modification or inhibition of the TGF-βR has been described to improve NK cell activity in multiple studies [21,87,88]. Deletion of the TGF-βRII subunit enhanced both mTOR activity and NK cell cytotoxicity following IL-15 stimulation [21]. Burga et al., developed novel truncated TGF-βR variants in which the RII subunit was fused to DNAX-activation protein 12 (DAP12) or a synthetic Notch-like receptor coupled to RELA, ultimately rewiring the TGF-β pathway to promote NK cell activity instead of suppression [87]. Burga et al., observed improved NK cell survival and tumor eradication using modified NK cells sourced from umbilical cord blood in a murine xenograft model of TGF-β-secreting neuroblastoma [87]. Recently, Neviani and colleagues showed that the tumor suppressor microRNA (mIR)-186 can downregulate TGF-βRI and RII [88]. Furthermore, low mIR-186 in high-risk neuroblastoma patients directly correlated with low NK cell expression of NKG2D and DNAM-1. mIR-186 delivered to NK cells via lypopolyplex nanoparticles coated with anti-CD56 induced resistance to TGF-β-induced immunosuppression and improved effector functions following IL-15 stimulation [88]. Finally, antibody–drug conjugates (ADCs) are currently one of the fastest growing classes of anti-cancer therapeutics and have traditionally consisted of mAbs attached via a chemical linker to a cytotoxic payload directed towards a cancer-specific antigen [89]. Only recently has the potential for ADCs been explored beyond anti-cancer therapeutics for inflammatory/autoimmune diseases and infectious pathogens [90,91]. We propose that the engineering of an ADC that could deliver a TGF-βR smi directly to NK cells has the potential to prevent postoperative NK cell dysfunction and subsequent cancer recurrence. Successful perioperative therapy has enormous potential to reduce the burden of cancer worldwide.

**Figure 7:**
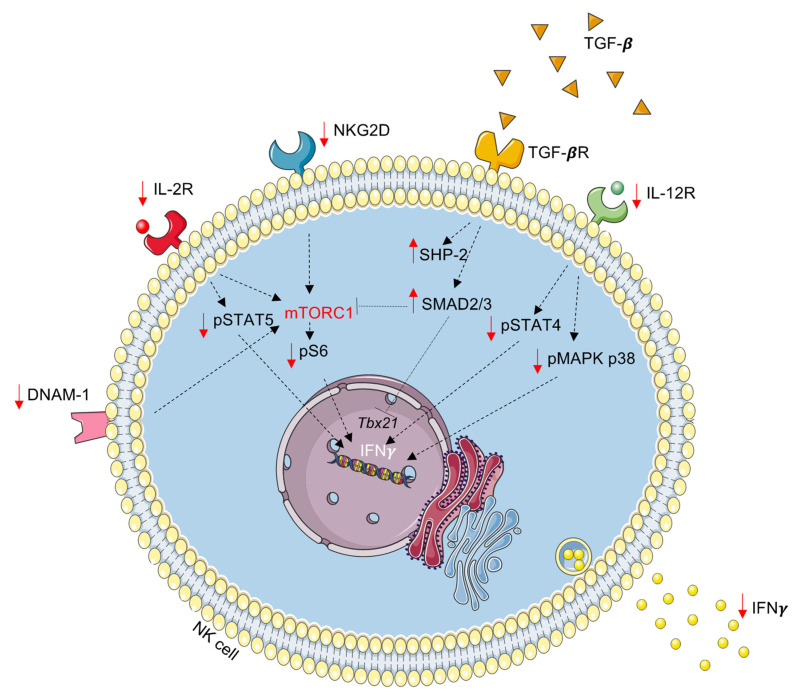
**Proposed mechanisms of TGF-β-mediated Natural Killer cell dysfunction.** Upon binding to its receptor, TGF-β may signal through SMAD2/3 to directly inhibit the expression of the transcription factor T-bet (*Tbx21*) or to inhibit mTOR via unknown intermediary protein(s). Smad-independent mechanisms may include the production and activation of SHP-2 phosphatase, which may indirectly mediate mTOR activity. These changes in intracellular signaling may then contribute to downregulation or activating receptors (IL-2R, IL-12R, NKG2D, DNAM-1). Finally, these phenotypic changes result in reduced cytokine (IFNγ) secretion and suppressed NK cell function.

To conclude, we report that surgical stress results in a significant reduction in NK cell surface expression of activating receptors, phosphorylation of downstream signaling proteins, and IFNγ production (significantly reduced also in the CD56^Bright^ population) in a cohort of patients undergoing cancer surgery (Figure 7). Notably, these effects could be reproduced in healthy NK cells when treated with recombinant TGF-β1 or postoperative patient plasma. Furthermore, NK cell single-cell RNA sequencing suggests increased activity of the TGF-β canonical (SMAD) and non-canonical signaling pathways (mTOR). Blocking TGF-β signaling in postoperative plasma was sufficient to restore NK cell phenotype and function. Finally, we assessed the activity of TGF-β canonical and non-canonical signaling pathways, revealing that postoperative NK cell suppression is mediated via SMAD2/3 signaling and the inhibition of mTOR. Together, this work highlights profound changes in postoperative NK cells and highlights a role for TGF-β as a potential therapeutic target in the perioperative period to enhance NK cell function and reduce the development of metastatic disease following cancer surgery.

### 4.1. Limitations

#### 4.1.1. Inter-Patient Variability

While healthy adults show very stable immune systems in the absence of perturbation, there is inherent heterogeneity and variability between patient samples [92,93,94]. Inter-patient variability is a consequence of both heritable factors, including host genetics and sex, and non-heritable factors, including the microbiota, bacterial dysbiosis, viral history, and other environmental factors, including the type and stage of cancer, in the case of the present study [95]. Juxtaposed with murine models that tout “unity in biology” through syngeneity, the inter-patient heterogeneity of humans provides challenges for data interpretation. Moreover, human systems do not allow for the same level of manipulation as murine models. However, the significant advantage over murine models is that studies may be more clinically relevant. Despite inter-patient variability in the targets we assessed, we observed universal changes in postoperative NK cell phenotype and function across a variety of types of cancer and surgery, which ultimately lends validity to these results.

#### 4.1.2. Measuring Soluble TGF-β

Although numerous studies have previously quantified changes in postoperative TGF-β, the plasma concentration of TGF-β in patients undergoing cancer surgery on POD1 had not been reported to our knowledge [15,96]. We assessed plasma TGF-β immediately pre-operatively at baseline and on POD1 and observed a trend toward increased TGF-β on POD1. However, this did not reach statistical significance. It is entirely possible that TGF-β levels peak earlier near the induction of surgical stress. Kato et al., reported that IL-10 levels peaked at 4 h during surgery and IL-6/IL-8 levels peaked at the end of surgery [96]. This is supported by work by Viel and colleagues who described an early mTOR-dependent inhibition of NK cells that may contribute later to inhibition of proliferation [21]. Future amendments to the blood collection timepoints in our clinical protocol will enable measurement of TGF-β at earlier timepoints in the postoperative period.

## 5. Conclusions

The immunosuppressive effects of surgery influence the development of postoperative complications, most notably infection [97]. For patients undergoing cancer surgery, the stakes are even higher, with immunosuppression presenting an opportunity for cancer cells to form metastases. However, this is also a window of opportunity in which to intervene. There are currently no immune-modulating therapeutics targeted to the perioperative period. We and others have shown that NK cells are functionally suppressed in the postoperative period and that this suppression contributes to the development of metastatic disease. Specifically, IFNγ production by NK cells is profoundly suppressed postoperatively and is associated with phenotypic perturbations in receptor expression and critical signaling molecules. Knowledge of the postoperative NK cell phenotype and mechanisms responsible for the induction of immune dysfunction is critical for the development of perioperative immunotherapies. Since ancient times, we have known that, despite the complete removal of a cancerous tumor, the disease may return [98]. Prevention of immune suppression is vital to the prevention of cancer recurrence. Here we present surgery-induced TGF-β1 as a potential target for the development of a novel perioperative therapeutic.

## Figures and Tables

**Table 1:** FACS monoclonal antibodies.

Antibody	Vendor	Cat #	Clone
CD3 FITC (mouse)	Invitrogen	11-0039-41	HIT3a
CD56 BV421 (mouse)	BD biosciences	562751	NCAM16.2
CD16 BV650 (mouse)	BD biosciences	563692	3G8
CD14 APC-Cy7 (mouse)	BD biosciences	557831	MφP9
CD45 AF700 (mouse)	BD biosciences	560566	HI30
Fixable viability dye BV510	BD biosciences	564406	-
IFNγ APC (mouse)	Invitrogen	17-7319-82	4S.B3
CD25 PE-Cy7 (mouse)	BD biosciences	557741	M-A251
CD122 PE (mouse)	BD biosciences	554522	Mik-β2
CD132 APC (rat)	Biolegend	338607	TUGh4
p-STAT5 PE-Cy7 (pY694) (mouse)	BD biosciences	560117	47/Stat5
CD212 BV786 (mouse)	BD biosciences	744207	2.4E6
p-STAT4 PE (pY693) (mouse)	BD biosciences	558249	38/p-Stat4
NKG2D BV650 (mouse)	BD biosciences	563408	1D11
NKG2A PE (mouse)	R&D Systems	FAB1059P-025	131411
PE-Cy7 DNAM-1 (mouse)	BioLegend	338315	11A8
APC TIGIT (mouse)	BioLegend	372705	A15153G
PD-1 PerCP-Cy5.5 (mouse)	BioLegend	329913	EH12.2H7
S6 PE (pS235/236) (mouse)	BD biosciences	560433	NF-548
p38 MAPK APC (pThr180, Tyr 182) (mouse)	Invitrogen	17-9078-42	4NIT4KK
Smad2(pS465/pS467)/Smad3(pS423/pS425) PE (mouse)	BD biosciences	562586	O72-670
Mouse PE IgG2a	BioLegend	400214	MOPC-173
Mouse APC IgG2A	BioLegend	400219	MOPC-173
Mouse PerCP-Cy5.5 IgG1	BioLegend	400149	MOPC-21
Mouse APC IgG1	Biolegend	400119	MOPC-21
Mouse PE-Cy7 IgG1	BD biosciences	557872	MOPC-21
Mouse PE IgG2b	Invitrogen	12-4732-41	eBMG2b
Mouse BV650 IgG1	BD biosciences	563231	X40
Rat APC IgG2b	Biolegend	400611	RTK4530
Mouse BV786 IgG1	BD biosciences	563330	X40

## Data Availability

The datasets for this study can be made available upon reasonable request.

## Supplementary Materials

The following supporting information can be downloaded at: https://www.mdpi.com/article/10.3390/ijms232314608/s1.
