# Supplementary material for: Preventing Surgery-Induced NK Cell Dysfunction Using Anti-TGF-β Immunotherapeutics
Source: Int J Mol Sci. 2022 Nov 23;23(23):14608. doi: 10.3390/ijms232314608 (PMC9737532; doi:10.3390/ijms232314608)
Supplement: Supplementary file 1 [file ijms-23-14608-s001.zip › ijms-1989251-supplementary.pdf]

Supplemental Data:

Supplemental Table S1. Demographics for whole blood samples used to characterized cytokine and activating/inhibitory receptors, phospho-signaling proteins, and intracellular/extracellular IFNγ.

Category	**Subcategory**	**Healthy Donors**	**Cancer Patients**
Total (n)		42	39
Sex	Male	16	21
	Female	26	18
Patient Age	< 60 years	26	11
	60-69 years	9	19
	> 70 years	7	9
Cancer Type	Prostate	-	11
	Lung	-	7
	Colorectal	-	6
	Renal	-	3
	Ovarian	-	3
	Sarcoma	-	3
	Pancreatic	-	1
	Parathyroid	-	1
	Neuroendocrine	-	1
	Endometrial	-	2
	Duodenal	-	1
Staging	I	-	14
	II	-	7
	III	-	15
	IV	-	3

Supplemental Table S2. Demographics for healthy CD56^{+} donors and healthy/cancer patient plasma used in plasma culture experiments.

Category	**Subcategory**	**Healthy NK Cell Donors**	**Healthy Plasma Donors**	**Cancer Patient Plasma**
Total (n)		18	27	58
Sex	Male	5	12	35
	Female	13	14	23
	Other	-	1	-
Patient Age	< 60 years	14	18	15
	60-69 years	3	4	24
	> 70 years	1	5	19
Cancer Type	Prostate	-	-	16
	Endometrial	-	-	10
	Lung	-	-	8
	Sarcoma	-	-	4
	Renal	-	-	2
	Colon	-	-	3
	Pancreatic	-	-	2
	Rectal	-	-	1
	Thymoma	-	-	1
	Hepatic	-	-	2
	Gastric	-	-	2
	Duodenal	-	-	2
	Non-cancer	17	26	3
	Inconclusive pathology	-	-	2
Staging	I	-	-	13
	II	-	-	9
	III	-	-	22
	IV	-	-	4
	Unknown	-	-	10

Supplemental Table S3. **Demographics for patient platelet-free plasma used to quantify extracellular TGFβ.**

Category	**Subcategory**	**Cancer Patient Plasma**
Total (n)		25
Sex	Male	17
	Female	8
Patient Age	< 60 years	5
	60-69 years	14
	> 70 years	6
Cancer Type	Prostate	8
	Lung	6
	Sarcoma	2
	Renal	2
	Endometrial	2
	Colon	2
	Hepatic	1
	Thymoma	1
	Breast	1
Staging	I	8
	II	5
	III	8
	IV	-
	Unknown	4

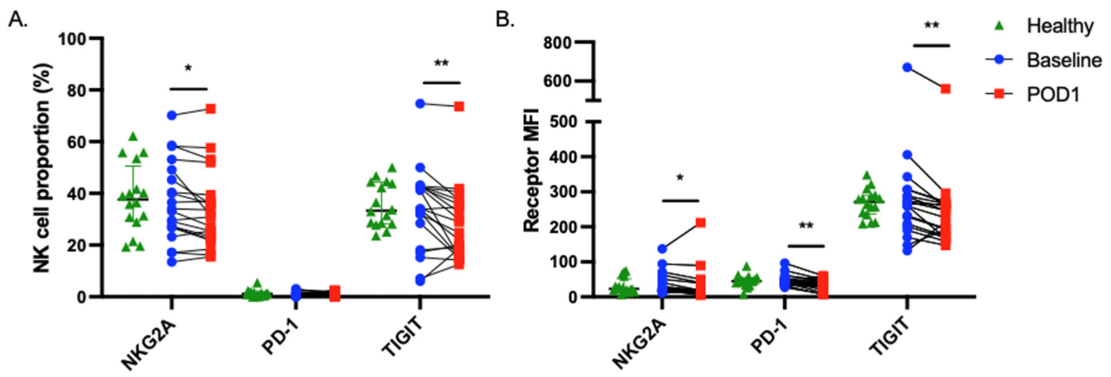

Supplemental Figure S1. Whole blood samples reveal changes in inhibitory receptors on POD1. Expression NKG2A (% and MFI), PD-1 (MFI), and TIGIT (% and MFI) were significantly changed on POD1 (A (%)/B (MFI)). ($^{\#}p\leq0.1$, $*p\leq0.05$, $**p\leq0.005$, $***p\leq0.0005$, $****p\leq0.00005$)
